# Menstrual pattern among adolescent girls of Pokhara Valley: a cross sectional study

**DOI:** 10.1186/s12905-016-0354-y

**Published:** 2016-12-09

**Authors:** Sudha Sharma, Sajana Deuja, C. G. Saha

**Affiliations:** Department of Physiology, Manipal College of Medical Sciences, Deep Height, P.O. Box 155, Pokhara, Nepal

**Keywords:** Adolescents, Dysmenorrhoea, Menarche, Menstruation, Menstrual pattern

## Abstract

**Background:**

Menstruation is a normal physiological process occurring every month throughout the reproductive age of the females. However, significant variation in menstrual pattern is observed among adolescents. The objective of this study was to determine menstrual pattern among adolescent girls of Pokhara Valley.

**Method:**

A cross sectional study was conducted in seven schools of Pokhara Valley. A total of 260 adolescent girls were included in the study. Girls were requested to complete questionnaire containing 19 items. Selected girls voluntarily agreed to answer questionnaire under the supervision of the researcher and then the data was analyzed.

**Results:**

Mean age of the girls was 14.0 ± 1.3 years. Mean age at menarche was 12.2 ± 0.9 years. The mean cycle length of the subjects was 34.8 ± 11.8 days. It was observed that as many as 167(64.2%) girls had irregular menstrual cycle and significant association was found between regularity of menstruation and ethnicity. Seven (2.7%) girls had a menstrual cycle length shorter than 21 days, 60(23.1%) had cycle longer than 35 days and 193(74.2%) had a normal cycle length between 21 and 35 days. Majority 231(88.8%) had normal duration of menstruation. Dysmenorrhoea was reported by more than half of the girls and significant association was found between severity of dysmenorrhoea with school absenteeism and treatment needed.

**Conclusion:**

Dysmenorrhoea was the most common menstrual problem among adolescent girls. Girls’ school attendance was affected due to menstrual pain. Girls with severe dysmenorrhea needed medical treatment. Irregular menstruation was reported by more than half of the girls and significant association was found with the ethnicity.

**Electronic supplementary material:**

The online version of this article (doi:10.1186/s12905-016-0354-y) contains supplementary material, which is available to authorized users.

## Background

Adolescence is a period between childhood and adulthood, a transition phase marked by development in secondary sexual characteristics and reproductive maturity [1]. According to World Health Organization (WHO) ‘adolescence’ is a period between 10 and 19 years [2]. Menarche is defined as the first menstrual period, the most notable event during female puberty [3]. Menstruation is a monthly endometrial shedding leading to the discharge of blood from the uterus occurring in every 28 ± 7 days [4, 5]. The average menstrual bleeding lasts about 5 days, which sums up to females having approximately 67 months of menstruation throughout her reproductive age [6].

Though menstruation is a part of the normal reproductive cycle of females, variation in menstrual pattern is frequently observed among adolescents [7]. Dysmenorrhea is defined as pelvic pain occurring during menstruation with symptoms of headache, back pain, nausea, vomiting and diarrhea [8]. It is a major problem during menstruation, affecting females’ daily activities including school absenteeism. Adolescent girls visit physician with complaints of delayed, irregular, painful, and heavy menstrual bleeding [9].

There has been a dearth of documented research on pattern of menstruation among adolescent girls in Pokhara Valley. The above fact has created an insight in the researcher’s mind to conduct this study. Therefore, the purpose of this paper is to determine age at menarche and menstrual pattern among adolescent girls of Pokhara Valley. The present study may also act as important evidence for the implementation of reproductive health policies for adolescents in Nepal.

## Methods

A cross-sectional descriptive study was conducted among adolescent girls of 10–19 years from seven different government and private schools of Pokhara Valley.

Pokhara Valley is the second largest valley in Hilli region located in the western part of the Nepal with population of 264,991.

All female students of grades 5 to 10 were invited to participate in the study as they were ideal group to study menstrual pattern among adolescent girls. A total of 260 students participated in the study. They were selected by using purposive non- probability sampling technique. Girls who had primary amenorrhoea, who had not attained menarche at the time of study, with age <10 and >19 years and those who couldn’t remember the exact date of menarche and menstruation were excluded from the study. Exclusion criteria was applied before the data was taken.

Before conducting the research, girls were acquainted with the purpose and procedure of the research. They were also informed that they deserve right to withdraw from the study anytime. With assurance of confidentiality, they were requested to sign on English and Nepali version of written consent. For students who were under age of 16, a verbal consent was taken from their parents. Only girl participants were allowed inside the class where data was collected. Questionnaires including 19 items were presented in English and thoroughly explained in Nepali language to each of the participants. Ethical approval was obtained from the Ethics and Research committee, Manipal College of Medical Sciences, Pokhara.

The questionnaire covered general information about the girls’ including full name, address, age, education, ethnic group, marital status. In Nepal, Hill caste and Dalit are classified under caste group, Hill Ethnic group are classified under Janajati/Adibasi and Muslim are classified under other groups on the basis of region [10]. Questions related to menstruation covered: age at menarche, duration of menstruation, cycle length, regularity of menstruation, dysmenorrhea and its severity (mild, moderate and severe). They were also asked whether they needed treatment for dysmenorrhea and impact on school attendance due to menstrual problem.

Age at menarche was obtained by using recall method and for that girls were asked to remember the grade at which they started having period. Age at menarche was further classified into early menarche, medium menarche and delayed menarche. Early menarche the age of 12 years or less, medium menarche between 13 and 14 years and delayed menarche more than 14 years [11]. Menstruation was considered regular where duration of menstruation was 21–35 days, whereas, it was considered irregular where period was less than 21 days or more than 35 days.

Girls’ height was recorded with their buttocks and back of their head in contact with smooth vertical wall looking straight forward. While taking their weight, girls were instructed to stand on both feet in the center of weighing machine. These values were further used to calculate the Body mass index (BMI) using formula BMI = weight in Kg/height in m^2^. BMI was classified into four groups based on the cut off points recommended by World Health Organization.

Statistical analysis was performed using Microsoft Excel (Version 2013) and Statistical Package for Social Sciences (SPSS, version 17). All the variables were presented in frequency and percentage. Chi - square test was performed for testing statistical significance association among variables. The statistical significant association was considered when *p* < 0.05.

## Results

A total of 260 girls were analyzed. The girls involved in the study were aged between 10 and 19 years with the mean and standard deviation of 14.0 ± 1.3 years. Majority 113(43.5%) represent Hill ethnic group which was followed by 102(39.2%) Hill caste, 23(8.8%) Muslim (others) and 22(8.5%) Dalit. Hence, the Hill ethnic group represents one of the leading population of Pokhara valley. Out of all the girls under the study, maximum 259(99.6%) were unmarried and only 1(0.4%) was married (Table 1).

**Table 1:** Socio-demographic characteristics of adolescent girls in Pokhara Valley

Variables	No (%)
Age	
10–14 years	166(63.8%)
15–19 years	94(36.2%)
Grade	
Grade 5	3(1.2%)
Grade 6	15(5.8%)
Grade 7	53(20.4%)
Grade 8	85(32.7%)
Grade 9	56(21.5%)
Grade 10	48(8.5%)
Ethnic group	
Hill Ethnic group	113(43.5%)
Hill caste	102(39.2%)
Muslim (others)	23(8.8%)
Dalit	22(8.5%)
Marital status	
Unmarried	159(99.6%)
Married	1(0.4%)
School	
Private	210(80.8%)
Government	50(19.2%)

In the present study, the mean and standard deviation of age at menarche by recall method was found to be 12.2 ± 0.9 years ranging between 9 and 15 years. Mean cycle length of the girls was found as 34.8 ± 11.8 days with maximum being 120 days and minimum 20 days (Table 2).

**Table 2:** Mean and standard deviation of age at menarche and cycle length of adolescent girls in Pokhara Valley

Variables	Maximum	Minimum	Mean ± SD
Age at menarche	15	9	12.2 ± 0.9 years
Cycle length	120	20	34.8 ± 11.8 days

In the distribution of subjects by onset of menarche, largest number of girls 164 (63.1%) had early menarche followed by 95 (36.5%) with medium menarche and only 1 (0.4%) had delayed menarche.

The menstrual pattern of subjects is shown in (Table 3) with respect to cycle length, duration of menstruation and regularity. Out of the total study population, 7(2.7%) girls had a menstrual cycle length shorter than 21 days, 60(23.1%) had cycle longer than 35 days and 193(74.2%) had a cycle length between 21 and 35 days. It was also observed that the majority 231(88.8%) had normal duration menstrual bleeding (3–7 days), 15(5.8%) experienced longer duration of menstrual bleeding with more than 7 days and 14(5.4%) reported duration of menstrual bleeding less than 3 days. Irregular cycle was experienced by 167(64.2%) girls whereas 93(35.8%) had regular cycle.

**Table 3:** Menstrual pattern of adolescent girls of Pokhara Valley

Menstrual pattern	No(%)
Cycle length	
< 21 days	7(2.7%)
21–35 days	193(74.2%)
> 35 days	60(23.1%)
Duration of menstruation	
< 3 days	14(5.4%)
3–7 days	231(88.8%)
> 7 days	15(5.8%)
Regularity	
Regular	93(35.8%)
Irregular	167(64.2%)

The regularity of menstruation is shown in (Table 4) with respect to age, school, ethnicity and BMI. There was a statistical significant difference on menstrual cycle regularity by ethnicity (*p* < 0.05). But, no statistically significant value of the regularity of menstrual cycle was found by age, school and BMI.

**Table 4:** Factors affecting regularity of menstruation in adolescent girls in Pokhara Valley

Variables	Regularity			
	Irregular (%)	Regular (%)	*X* ^2^	p value
Age				
10–14 years	113(68.1%)	53(31.9%)	2.94	0.086
15–19 years	54(57.4%)	40(42.6%)		
School				
Private	140(66.7%)	70(33.3%)	2.82	0.093
Government	27(54%)	23(46%)		
Ethnicity				
Hill caste	61(59.8%)	41(40.2%)	12.10	0.007
Hill Ethnic group	81(71.7%)	32(28.3%)		
Dalit	8(36.4%)	14(63.6%)		
Muslim (Others)	17(73.9%)	6(26.1%)		
BMI				
Underweight	63(59.4%)	43(40.6%)	2.25	0.521
Normal	85(68%)	40(32%)		
Overweight	11(61.1%)	7(38.9%)		
Obese	8(72.7%)	3(27.3%)		

Dysmenorrhoea was reported by 186(71.5%) subjects, of which, 99 (53.2%) had mild, 70 (37.6%) had moderate and 17 (9.1%) had severe dysmenorrhea. The result showed the highly significant association between school absentees and severity of dysmenorrhea (*p* < 0.001). However, regularity of menstruation was not significantly associated with the severity of dysmenorrhea (*p* > 0.05). Similarly, highly statistical significant association was found in between treatment needed for dysmenorrhea and severity of dysmenorrhoea (*p* < 0.001) (Table 5).

**Table 5:** Factors associated with severity of dysmenorrhea

Variables	Severity of dysmenorrhea					
	Mild	Moderate	Severe	Total	*X* ^2^	p value
	*n* = 99	*n* = 70	*n* = 17	*n* = 186		
Regularity						
Regular	65(52.4%)	46(37.1%)	13(10.5%)	124	0.80	0.667
Irregular	34(54.8%)	24(38.7%)	4(6.5%)	62		
Absentees						
Yes	21(21.4%)	42(53.6%)	15(25%)	78	41.80	*p* < 0.0001
No	78(71.6%)	28(26.6%)	2(1.8%)	108		
Treatment needed						
Yes	1(4.8%)	4(19%)	16(76.2%)	21	1.29	*p* < 0.0001
No	98(59.4%)	66(40%)	1(0.6%)	165		

## Discussion

The age at menarche in our study was consistent with the earlier studies conducted in the Pokhara valley [12] and in other parts of Africa and world [13–15]. However, the result differed from the study conducted in Ethiopia, where mean age at menarche was found as 15.8 ± 1 years [11]. Precision in this data might have affected as girls were asked to give whole year regarding age at menarche.

Distribution of subjects by onset of menarche shows that, largest number of girls 164 (63.1%) had early menarche followed by 95 (36.5%) had medium menarche and only 1 (0.4%) had delayed menarche. This result was contrast to the study done by Ali AA et al. [16] in which maximum 502 (76.4%) had late menarche followed by 127 (19.3%) medium menarche and the least 28 (4.3%) experienced early menarche. Study suggests that girls with early menarche are more prone to alcohol abuse and early sexual initiation [1]. Hence, there is a need of awareness and sex education in order to prevent them from sexually transmitted disease and unwanted pregnancy during their adolescence.

The mean cycle length of the girls in our study was 34.8 days, with standard deviation of ±11.8. The result obtained suggests that, the mean cycle length of the subjects in our study is higher than the values obtained in the other studies done in India, Ghana and Turkey where mean cycle length were found to be 28.7 ± 3.26 days, 27.9 ± 0.9 days, 27.7 ± 2.5 days respectively [13, 17, 18]. This can be explained by higher mean age of girls in those studies compared to this. Within the earlier years after the menarche menstrual cycle length tends to be longer due to immaturity in the hypothalamic–pituitary–ovarian axis. Cycle length becomes normal ranging between 21 and 34 days after third gynaecological year in 60–80% cycles [19].

The results on cycle length obtained by Rigon F et al. [14] shows that 3% of girls reported menstrual cycle shorter than 21 days that is comparable to our study 7(2.7%) whereas, only 3.4% experienced menstrual cycle longer than 35 days which is far less than finding in our study 60(23.1%). Similar studies done in Malaysia [9] and Sudan [16] shows variable results regarding cycle length.

The result obtained about duration of menstruation showed that, majority 231(88.8%) reported normal duration of menstruation (3–7 days) which is comparable to the value obtained by Pitangui AC et al. [20] in their study. A careful attention must be given for those having longer menstrual bleeding as the study suggests that longer menstrual bleeding could lead to iron deficiency anemia [14].

Irregular menstrual period was reported by more than half of the girls involved in the study. Different studies reported varying result regarding menstrual cycle irregularity among adolescent girls. Ali AA et al. [16] observed it in 31.5%, Patil SM et al. [21] in 7.5%. This disparities could be due the difference in the selection of study population. Among 167 of girls with irregular cycle, only 17(10.1%) girls were within the two years after attaining menarche. Irregular menstruation is normal phenomenon among females within the two years of menarche [11]. More number of girls with irregular cycle in our study could be due to the higher percentage of young girls aged between 10 and 14 years, as study suggest that normal cycle length is obtained around the chronological age of 19–20 [19].

The result of the present study showed a significant association between ethnicity and regularity of menstruation (*p* < 0.0001). Relatively, more percentage of Dalit girls had regular menstrual cycle due to the fact that maximum number 20(90.9%) of dalit girls were farthest away from the menarche. This could have resulted in the difference seen in regularity of cycle among ethnic groups.

Dysmenorrhoea was the major menstrual problem among adolescent girls and was reported by 186(71.5%) of the girls with varying degree of severity. This figure was comparable to 73% reported by Pitungi AC [20] and 72% by Zegeye DT et al. [11] in their studies.

Severity of dysmenorrhea was significantly associated with school absence in adolescent girls (*p* < 0.0001). This finding is consistent with the result obtained by Zegeye DT et al. [11] and Cakir M et al. [18] in their studies. This indicates the serious problem in adolescent girls as many study has reported decline in academic performance due to dysmenorrhea in females [22, 23]. The statistical significant association was found in between treatment needed for dysmenorrhea and severity of menstruation (*p* < 0.0001). Most of the adolescent with dysmenorrhoea follow self-medication practice with few consulting health care provider [24].

A study done by Zegeye DT et al. showed a link between regularity of menstruation and severity of dysmenorrhea [11]. However, this study did not find any significant association between severity of dysmenorrhoea and regularity of menstruation which could be due to small sample size of this study.

A potential limitation of our study is recall bias, as all of the variable related to menstruation were taken by recall method. Also, there were more number of girls between 10 and 14 years, who may not show post-menarche menstrual pattern. Memory bias about menarche can be neglected as menarche is very notable event considering its physiological and cultural perspective, as there is a custom of isolating girls during their first period in Nepal.

In this context, the data obtained in our study could be a guide to the government bodies for the implementation of a proper health policy addressing the reproductive health of the adolescent girls of Nepal.

## Conclusion

Dysmenorrhea was the most common menstrual disorder and has significantly affected the school attendance. Girls with severe dysmenorrhea needed medical treatment. More than half percentage of adolescent girls of Pokhara Valley reported irregular menstruation. Regularity of menstruation significantly varied with the ethnicity. Further studies could be done to determine reasons for the disparities occurring among different ethnic groups regarding menstrual regularity (Additional file 1).

## Additional file

Additional file 1: Questionnaire. Questionnaire containing socio-demographic profile and menstrual characteristics of adolescent girls. (DOCX 33 kb)

## Abbreviations

BMI: Body mass index
Kg: Kilogram
m: Meter
SD: Standard deviation
SPSS: Statistical package for social sciences
WHO: World Health Organization
