# Supplementary material for: Menstrual pattern among adolescent girls of Pokhara Valley: a cross sectional study
Source: BMC Womens Health. 2016 Dec 9;16:74. doi: 10.1186/s12905-016-0354-y (PMC5148896; doi:10.1186/s12905-016-0354-y)
Supplement: Additional file 1: — Questionnaire. Questionnaire containing socio-demographic profile and menstrual characteristics of adolescent girls. (DOCX 33 kb) [file 12905_2016_354_MOESM1_ESM.docx]

**Questionnaire**

1. **General Information:**

Name: Age: Address:

Ethnic Group: Education level:

School:

Type of School: Government Private

Height (kg): Weight (m):

Marital status: Married Unmarried

1. **Information about Menstruation:**

Mean age at menarche (age at which first menstruation started):

Cycle length (Number of day from first day of your last menstrual period to last cycle day before you start menstruating again):

Cycle length:

1. 21-35 days
2. Less than 21 days
3. More than 35 days

Duration of menstruation (days): Number of days bleeding last

1. 3-7 days
2. Less than 3 days
3. More than 7 days

Regularity of cycle:

1. Regular
2. Irregular

Pain during menstruation : Yes No

If yes, Mild Moderate Severe

Treatment needed for pain: Yes No

School absentees due to pain: Yes No
